# Characterization of HIV-1 Near Full-Length Proviral Genome Quasispecies from Patients with Undetectable Viral Load Undergoing First-Line HAART Therapy

**DOI:** 10.3390/v9120392

**Published:** 2017-12-19

**Authors:** Brunna M. Alves, Juliana D. Siqueira, Marianne M. Garrido, Ornella M. Botelho, Isabel M. Prellwitz, Sayonara R. Ribeiro, Esmeralda A. Soares, Marcelo A. Soares

**Affiliations:** 1 Programa de Oncovirologia, Instituto Nacional de Câncer, Rio de Janeiro 20231-050, Brazil; brunna_alves@yahoo.com.br (B.M.A.); sidoju@hotmail.com (J.D.S.); ornellamartins@hotmail.com (O.M.B.); imprellwitz@gmail.com (I.M.P.); esoares@inca.gov.br (E.A.S.); 2 Serviço de Doenças Infecciosas, Hospital Federal de Ipanema, Rio de Janeiro 22411-020, Brazil; marianne.monteiro@inca.gov.br (M.M.G.); sayorib@gmail.com (S.R.R.); 3 Departamento de Genética, Universidade Federal do Rio de Janeiro, Rio de Janeiro 21944-970, Brazil

**Keywords:** HIV-1, quasispecies, minority resistance mutations, HAART, drug resistance, undetectable viral load

## Abstract

Increased access to highly active antiretroviral therapy (HAART) by human immunodeficiency virus postive (HIV^+^) individuals has become a reality worldwide. In Brazil, HAART currently reaches over half of HIV-infected subjects. In the context of a remarkable HIV-1 genetic variability, highly related variants, called quasispecies, are generated. HIV quasispecies generated during infection can influence virus persistence and pathogenicity, representing a challenge to treatment. However, the clinical relevance of minority quasispecies is still uncertain. In this study, we have determined the archived proviral sequences, viral subtype and drug resistance mutations from a cohort of HIV^+^ patients with undetectable viral load undergoing HAART as first-line therapy using next-generation sequencing for near full-length virus genome (NFLG) assembly. HIV-1 consensus sequences representing NFLG were obtained for eleven patients, while for another twelve varying genome coverage rates were obtained. Phylogenetic analysis showed the predominance of subtype B (83%; 19/23). Considering the minority variants, 18 patients carried archived virus harboring at least one mutation conferring antiretroviral resistance; for six patients, the mutations correlated with the current ARVs used. These data highlight the importance of monitoring HIV minority drug resistant variants and their clinical impact, to guide future regimen switches and improve HIV treatment success.

## 1. Introduction

According to the Joint United Nations Programme on HIV/AIDS, approximately 36.7 million people were living with the human immunodeficiency virus (HIV) worldwide at the end of 2016, making HIV infection a major public health problem [1]. One of the factors related to the increased number of people living with HIV is the greater access to highly active antiretroviral therapy (HAART), which is strongly associated with an increase in the life expectancy and quality of this population. By 2016, 19.5 million people had access to HAART, an increase of 6% over 2015 [2]. In Brazil, it has been estimated that about 830,000 individuals were living with HIV/AIDS by the end of 2016, representing an HIV prevalence rate of 0.24%. The access to HAART reached approximately 490,000, which represents more than half of the estimated Brazilian HIV-positive individuals. Of this total, approximately 450,000 had an undetectable viral load at least six months after HAART initiation, one indicator of therapeutic success [1]. According to the Brazilian Ministry of Health, virologic failure occurs when HAART fails to suppress and sustain a person’s undetectable viral load after six months of initiating or modifying treatment, in addition to the detection of viral load in patients who were previously undetectable [3]. Brazil has been cited as a reference in access to HAART since November 1996, when the government guaranteed universal and free access to therapy to the Brazilian HIV^+^ population. New recommendations that stimulate the initiation of HAART for all HIV-positive individuals, independent of CD4^+^ T lymphocyte counts, were implemented in October 2013 in order to reduce the transmission of the virus [4].

A remarkable HIV-1 genetic variability results from mutational events associated with the high error-prone rate of the viral reverse transcriptase (RT), high virus replication rates and homologous recombination events. HIV-1 diversity imposes an important clinical challenge, as it allows the virus to adapt to and evade immune responses and antiretroviral therapy [5], therefore influencing diagnosis and treatment [6]. In the context of continuous HIV-1 genetic variability within each individual, several highly related but genetically distinct variants are generated and referred to as viral quasispecies [7]. The quasispecies heterogeneity associated with the selective pressure exerted by the immune system influences virus persistence and pathogenicity, allowing the adaptation of the quasispecies through intrahost persistence and the ability to outgrow other less adapted variants [8].

The HIV evolutionary dynamics associated with ART pressure allows the appearance of drug-resistant variants [9,10]. Antiretroviral resistance is an important public health concern since it limits therapeutic options, causes treatment failure and can be transmitted, compromising future treatment option in untreated individuals [11,12]. Rates ranging from 4 to 14% of newly diagnosed patients have been reported in different parts of the world to be infected with strains that have at least one transmitted drug resistance mutation (TDRM) [13,14,15,16].

Aiming to establish more efficient HAART regimens, genotypic assays to detect drug resistance-associated mutations have been widely used in the clinical setting [17]. This practice has lately been greatly benefited by the use of Next-Generation Sequencing (NGS), which provides a large data volume in a cost-effective and highly sensitive way. Of note, NGS allows the detection of HIV minority variants, previously undetectable by Sanger sequencing, which has a detection limit of 10–25% frequency in the viral population [18,19,20]. Recent studies using NGS for HIV-1 sequencing were able to detect minority variants below 1% in frequency in the viral population, allowing the identification of drug-resistant minority variants, the study of transmitted resistant viruses and the impact of those minority variants on treatment efficacy [21,22,23,24,25]. Moreover, HIV-1 sequencing by NGS significantly contributes to the analysis of viral genetic diversity, evolutionary and epidemic processes, since near full-length genomes (NFLG) can be obtained [26]. These genomes contribute to the growing interest in tests that simultaneously probe multiple genomic regions that are targetted by antiretroviral drugs acting on different steps of the virus replicative cycle [27].

HIV minor drug resistant variants may persist in the infected individual [28,29]. Despite their low replicative capacity due to the presence of drug-resistance mutations, they may persist as archived proviruses in peripheral blood mononuclear cells (PBMCs) for several years and may have a long-term impact on therapeutic response [30,31,32]. However, the clinical significance of these minority drug resistant variants is still uncertain, as their role in the future response to treatment is not fully understood. Some studies have shown the association of such variants with increased risk of treatment failure in treated patients, as well as in patients with no previous ART history [21,33,34,35,36,37,38]. On the other hand, different studies did not find any influence of these minority resistant variants on treatment response [39,40,41,42,43,44,45]. The association between HIV minor non-nucleoside reverse transcriptase inhibitor (NNRTI) resistant variants and a worse treatment prognosis has been described [25,29,46,47,48]. Patients carrying those variants had a three-fold higher risk of treatment failure compared to patients without NNRTI-resistance mutations when subjected to therapeutic regimens based on this antiretroviral (ARV) class [49,50]. With respect to patients under suppressive therapy, a single study was conducted that showed the presence of minority drug-resistance mutations in five out of eleven patients and a large variability of the archived proviral epitopes [51]. This study highlights the importance of more sensitive genotypic resistance tests and further studies about the influence of these mutations on HAART outcomes, especially in patients under successful therapy, since this cohort is still scarcely discussed.

In the present study, we have assessed the presence of HIV minority drug-resistant variants archived in PBMC samples in a Brazilian cohort of chronic HIV patients undergoing HAART as first-line therapy and with undetectable viral load. Upon analyzing the HIV antiretroviral resistance profiles of the patients by NGS, our study was able to evidence a high prevalence of drug resistance mutations in this cohort, despite the therapeutic success achieved by their carriers. This is the first study investigating the presence of HIV minority drug resistance mutations among Brazilian patients under virologic control.

## 2. Materials and Methods

### 2.1. Study Population and Sample Collection

A cross-sectional study was carried out among patients attending a sexually transmitted diseases/HIV ambulatory at Hospital Federal de Ipanema, Rio de Janeiro, Brazil, between February and July 2016. These patients were recruited during the clinical follow-up routine and had a 10 mL sample of whole peripheral blood collected. A questionnaire was applied to collect epidemiological (date of birth, sex, risk behavior) and clinical (date of HIV diagnosis, HIV-1 viral load, CD4^+^ T-cell counts, CD8^+^ T-cell counts, treatment history) data. Written informed consent was obtained from all participants and data were processed using unique identifiers to ensure confidentiality.

Inclusion criteria included age ≥ 18 years, being under first-line HAART and having undetectable HIV viral load for at least 12 months prior to collection date. Patients who had a history of previous virological failure, that underwent a change in the therapeutic regimen due to intolerance or poor adherence, that were classified into clinical and/or immunological AIDS (had an AIDS defining disease or CD4^+^ counts equal or less than 200 cells/mm^3^, according to the U.S. Centers for Diseases Control and Prevention criteria, 2014) [52] or that were under follow-up at the reference center for less than 12 months were excluded from the analysis. By using those strict criteria, we have been able to enroll a total of 32 patients among the whole patients followed-up at that site. This research was approved on March 29th, 2016 by the Ethics Committees in Research of the Brazilian National Cancer Institute (INCA) and of Hospital Federal de Ipanema (CAAE 52862016.9.0000.5274).

### 2.2. DNA Extraction and PCR of Proviral DNA

Plasma and buffy coats were separated by centrifugation of the whole blood, and the latter were used for genomic DNA extraction with the Genomic DNA Extraction Kit (Real Genomics, BioAmerica, Inc., Miami, FL, USA) following manufacturer’s specifications.

Nested PCR was carried out for the amplification of near full-length HIV genomes using a set of five overlapping fragments of approximately 2 kb each, or alternatively four fragments, with ~3 kb each, as previously described [53,54]. Each fragment overlaped the adjacents by an average of 400 bp (minimum 89 bp, maximum 555 bp). All reactions were performed in a Veriti^®^ 96 Well Thermal Cycler (Life Technologies, Carlsbad, CA, USA) using Platinum™ Taq DNA Polymerase High Fidelity (Life Technologies) in a final volume of 25 µL. An overview of all PCR fragment coverage regions and list of primers used are provided in Table S1.

PCR products were visualized in 1% agarose gels. Duplicates were made for each PCR and pooled before proceeding to the purification step with the GFXTM PCR DNA and Gel Band Purification Kit (GE Healthcare, Chicago, IL, USA). The purified fragments were quantified in a NanoDrop ND 1000 apparatus (Thermo Scientific, Waltham, MA, USA) and diluted to 4 ng/µL before being pooled per sample. The final product pool per sample was diluted to 0.4 ng/µL for library construction.

### 2.3. Library Construction and NGS

Libraries were prepared using the Nextera XT DNA Sample Preparation kit (Illumina Inc., SanDiego, CA, USA) according to the manufacturer’s protocol. Briefly, after the fragmentation step using transposon technology, libraries were subjected to a PCR where they were tagmentated with Illumina sequencing adaptors and molecular tags (indexes) to identify each sample. A purification step was performed to select fragments of ~800 bp and libraries were quantified by qPCR with the KAPA library quantification kit (Kapa Biosystems, Wilmington, MA, USA). Individual libraries were then diluted to 4 nM, considering the mean size of each library and the quantification performed, and pooled. The final product was diluted to 12 pM and sequenced in a MiSeq Illumina platform (2 × 301 paired-end run) (Illumina) with 1% denatured PhiX DNA as a sequencing control.

### 2.4. Data Analysis

The analysis of the obtained files was performed in Geneious v.9.1.3 as described by Dudley et al. [55]. Briefly, the two fastq files generated per sample after the demultiplexing process were paired and trimmed at the ends to an error rate below 0.1%. The products were then used in the assembly of the viral genome sequence using an annotated HIV-1 HXB2 reference sequence made available by Dudley and colleagues, including information on resistance mutation-associated positions, genes, CDS and mature peptide positions (https://dholk.primate.wisc.edu/wiki/dho/public/page.view?name=default). The alignment parameters used in the assembly were as previously described [55]. For this alignment, ten iterations were used, where the first of the ten alignments was performed against the annotated reference sequence and the remaining nine used the consensus obtained in the previous step as reference, in order to reduce the influence of the reference used in the alignment product.

#### 2.4.1. Analysis of Resistance Mutations

The annotated drug resistance mutations were based on the consensus of the International Antiviral Society [56] and the Stanford HIV Drug Resistance Database (available at http://hivdb.stanford.edu/hiv/). Transmitted drug resistance mutations (TDRM) were also included and defined according to the classification of the World Health Organization established by Bennett et al. [11] and in the TDRM database of the Stanford HIV Database. The HIV-1 HXB2 reference annotation was manually updated to include new positions at the lists mentioned above and resistance mutations described in the literature at the C-terminal region of RT covering the connection (CN) and RNase H (RH) subdomains [57,58,59,60,61,62,63,64]. The mutations evaluated in the RT C-terminal region are listed in detail in Table 1.

The variant finder of Geneious v.9.1.3 was used to call nucleotide variants from the reference sequence at frequency higher than 1%. This tool evaluates nucleotide substitutions found in the alignment along with their frequency and whether they are synonymous or non-synonymous. The frequency and the number of reads representing each ARV-resistant variant was determined in relation to the annotated reference and exported to an Excel file. Variants between 20% and 1% of frequency were considered as minor variants, since 20% is the minimum frequency associated with detection of mutations by commercial genotype resistance assays that use Sanger sequencing [65].

#### 2.4.2. Phylogenetic Analysis

A consensus sequence was derived for each sample from the reference assembly with Geneious using the 50% stringency setting. HIV-1 subtype classification of each query sequence was inferred through phylogenetic analysis performed with the maximum likelihood (ML) method using PhyML v.3.0 [66] and the best model of nucleotide substitution was inferred with Model Generator [67]. Sequences suggestive of intersubtype recombination were further analyzed with the *bootscanning* tool of Simplot v.3.5.1 [68] for determining patterns of recombination and the HIV-1 subtypes involved in the recombination event. The following parameters were used: window = 400 pb; steps = 40 pb; T/t = 2.0; gapstrip = on; replicas = 100; nucleotide substitution model = F84; method = Maximum Likelihood. Recombinant strains were further confirmed by phylogenetic analysis of individual HIV-1 subtype genomic fragments as suggested by the *bootscanning* breakpoint analysis.

#### 2.4.3. Analysis of Viral Tropism

For the phenotypic prediction of HIV co-receptor usage, the reads spanning the complete region of V3 loop of the *env* gene were selected and unique haplotypes were identified and their relative frequency considered. Amino acid sequences of the haplotypes were analyzed only if started and ended with cysteine residues and contained 32–38 amino acid residues, a pattern consistent with functional sequences [69]. Valid haplotypes were analyzed with the Geno2Pheno algorithm (available at http://coreceptor.geno2pheno.org) [70] using a false positive rate cut-off of 10% for classification as R5- or non-R5-using viruses, as recommended by the European Consensus Group on Clinical Management of HIV-1 Tropism Testing and used in several studies [54,71,72,73,74].

## 3. Results

The median age of the patients included in the study was 38 years and 75% were male (Table 2). The median time of antiretroviral treatment at enrollment was approximately three years and the median baseline CD4^+^ T-cell count was 712.5 cells/mm^3^. All patients have reported being infected by sexual transmission, 56% of which were men who have sex with men. Most patients (19; 59%) were under HAART composed of tenofovir (TDF), lamivudine (3TC) and efavirenz (EFV) at the time of sample collection, but less frequent ARV schemes were also used (Table 3).

Nine samples (28%) failed to have more than one viral DNA fragment PCR-amplified and were excluded from further analyses, while 23 samples amplified at least two fragments and were sequenced using NGS. Of the latter, eleven samples had the near full-length genome (NFLG) sequenced, and the remaining samples were missing one or two DNA fragments over the genome. The average number of reads obtained per sample were 774,536 (374,780–1,647,090). After assembling, the average coverage per nucleotide position was 7193 and it was homogeneous over the regions sequenced. The Gag CDS was complete for 22 samples (96%), the Pol CDS for 15 (65%) and the Env CDS for 17 (75%).

Most samples (18; 78%) carried archived viruses harboring at least one mutation associated with antiretroviral resistance (Table 3). Among them, 13 samples had mutations in the RT region, including the connection domain. Four carried resistance only to NRTI, six only to NNRTI, while three presented resistance mutations to both NRTI and NNRTI. The ART regimen of these 13 patients inlcuded two NRTI and one NNRTI. One sample (patient #28) presented four thymidine analogue-associated mutations (TAMs), with frequency varying from 38.7 to 99.5%, that are associated with intermediate resistance to TDF, part of the patient’s current therapy regimen. Three samples presented viruses harboring the M184V mutation (frequency varying from 1.3 to 8%) that confers high-level resistance to 3TC, used by those patients. However, this mutation is known to increase the susceptibility to zidovudine (AZT) and TDF, that were also part of their ART regimen. Two samples presented the E399G mutation with frequency of 1.5% and 4.9%. This mutation is associated with resistance to EFV which was included in these patients’ therapy. Tree samples presented major resistance mutations in the protease sequence, and while two of them used protease inhibitors (PI), the mutations observed were not related to the PI used. Resistance mutations in the integrase region were found in five samples. Most of them were minority variants, present in frequency below 11%, while one (T66I) was found with frequency of 31% in patient #29. Only one mutation associated with resistance to entry inhibitors was found in the *env* gene, with a frequency of 3% in patient #13.

Follow-up data of the patients under study (i.e., HIV viral load and CD4^+^ T-cell counts) were obtained during the preparation of this report to evaluate the clinical progress of the viral infection. The time information varied between 9 and 17 months after the initial collection time. Median baseline CD4^+^ T-cell counts were 758 cells/mm^3^ (*n* = 31; IQR_50_ 715–963) and median baseline CD8^+^ T-cell counts were 8675 cells/mm^3^ (*n* = 25; IQR_50_ 657.75–1035), higher compared to the values observed at the time of patient enrollment. With respect to HIV viral load (*n* = 31), only one patient presented a detectable load (patient #8; 222 copies/mL) approximately one year after inclusion in the study. There was no change in the antiretroviral regimen of any patient, except for patients using LPV/r, who were switched to DRV/r due to changes in first-line regimens recommended by the Brazilian Ministry of Health.

**Figure 1:**
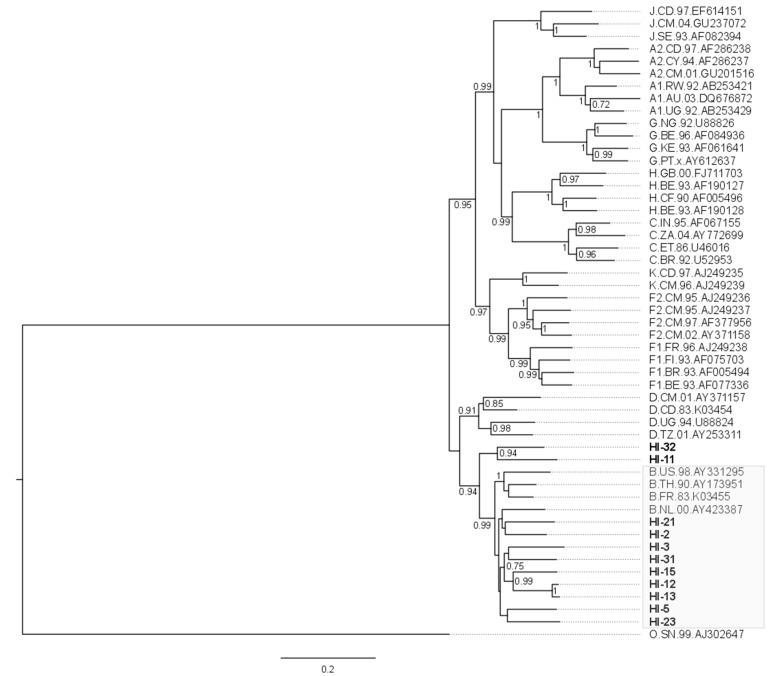
Phylogenetic maximum likehood analysis of HIV near full-length genomes obtained in this study. The analysis was conducted with 1000 bootstrap iterations and included eleven HIV-1 proviral sequences from Hospital Federal de Ipanema, Rio de Janeiro (represented in bold) and references of HIV-1 subtypes (represented by the subtype, country, year and GenBank accession number). Only bootstrap values greater than 0.7 are shown. The gray box highlights the sequences classified as subtype B, among which nine of the eleven sequences were placed. The two remaining sequences represented unique recombinant forms comprising subtype B and other subtypes.

**Figure 2:**
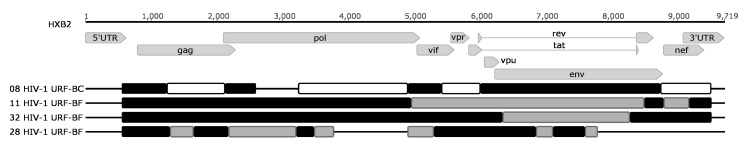
Phylogenetic classification of recombinant viruses considering the phylogeny and similarity analyses. The gray shading patterns represent the different subtypes: black for subtype B, gray for subtype F1 and white for subtype C. Sample IDs are represented at the left of each virus structure, which is in-scale relative to the genomic coordinates of the reference HXB2 genome at the top.

The HIV-1 consensus sequence was generated for each sample for phylogenetic analysis. Most NFLG obtained were from HIV-1 subtype B (*n* = 9; 82%; Figure 1). The two remaining were classified as distinct unique recombinant forms (URF) comprising subtypes B and F1 based on Simplot analysis (Figure 2). The sequences from the twelve non-complete genomes were predominantly of subtype B (*n* = 10), and two URF were found, one URF-BF1 and one URF-BC (Figure 2).

Two of the 11 NFLG described harbored stop codons in one or more open reading frames (ORF). One (patient #5) had multiple stop codons in the *gag* and one in the *pol* gene, consistent with APOBEC-mediated hypermutation (G→A changes), yet the number of substitutions did not allow the classification of this sequence as hypermutated. The other (patient #15) had a frameshift deletion (7 bp) within the *pol* gene, generating multiple stop codons in that ORF.

Virus envelope tropism analysis was performed with the reads spanning the V3 *env* region of the sequences and the resutls are depicted in Table 3. All but one sample contained the V3 loop region sequenced and were analyzed. We found that 17 (77%) patients showed CC chemokine receptor 5 (CCR5) usage as the major tropism profile (R5), while 5 patients (23%) presented predominantly the CXC chemokine receptor 4 (CXCR4) tropism profile (X4 and/or R5X4).

## 4. Discussion

Several viral factors are known to affect and modulate progression of HIV-1-related AIDS from the early assymptomatic phase of the disease, like the virus genetic diversity, viral fitness and co-receptor tropism [75,76,77,78,79]. In this context, we analyzed the archived HIV-1 proviral sequences from HIV patients in the early chronic phase of infection under HAART and with undetectable HIV viral load through NGS. This type of population is poorly explored in high-throughput studies, despite being of great relevance due the increased access to HAART worldwide. In the present report, we describe the genetic variability, prevalence of drug resistance-associated mutations and viral tropism of those patients at an ultra-deep level. It is noteworthy that this is the first study investigating the presence of minority drug resistance mutation among Brazilian patients under virologic control.

The majority of our patients self-reported as men who have sex with men (56%), had a median time of HIV-1 diagnosis to initiation of ART of about 1.2 years and they have been successfully treated for approximately three years. With the new recommendations by the Brazilian Ministry of Health implemented at the end of 2013, it is expected that the time lapse between HIV diagnosis and HAART initiation approaches zero, which contributes to reducing virus transmission. On the other hand, the test-and-treat approach highlights the importance of monitoring the prevalence of antiretroviral resistance and TDRM at population level, to avoid resistance dissemination and therapeutic failures due to transmitted drug resistance.

As seen in Latin America and the Caribbean countries, our study found a prevalence of subtype B infections (19; 83%) [80,81,82]. Although subtype B is the major HIV-1 genetic clade circulating in the Brazilian epidemic, the overall prevalence of non-B strains, like URF_BF1, URF_BC, and particularly subtype C and CRF31_BC in the South of Brazil, is increasing [83,84,85,86,87,88,89,90,91,92]. None of the BF1 (*n* = 3, 13%) or the BC recombinant structure (*n* = 1, 4%) identified in this study showed similarity with the recombination patterns of known CRFs or other recombinants. CRFs were also not detected among HIV-1 NFLG obtained from children and adolescents from São Paulo, Brazil, which corroborate the hypothesis that novel recombinants are continually arising in the Brazilian epidemic [89]. The analysis of HIV-1 NFLG, as opposed to most previous Brazilian HIV subtype studies that analyzed HIV sequence information only from shorter fragments, may unveil an underestimation of the occurrence of recombinant viruses in the country.

The integrated state of HIV allows its persistence as archived proviruses in PBMCs. Consequently, this may potentially compromise the efficacy of targeted antiretroviral drugs exerting a long-term impact on responses to HAART [31,93]. Also, it should be noted that routine genotyping tests focus on plasma circulating variants and on a limited number of short genomic fragments, despite the increasing need to simultaneously probing multiple genomic regions that target different steps of the viral life cycle. These facts, coupled with previous observations that standard bulk sequencing cannot fully access the spectrum of viral variants archived in the proviral DNA, justify the use of NGS technologies to study proviral DNA from PMBC as a valuable source for resistance analysis [17,94,95,96].

We found 18 patients (78%) who were successful responders to first-line ART (undetectable HIV viral load) that carried archived viruses harboring at least one drug resistance-associated mutation. Only a single study has been previously published in this regard on HIV patients experiencing suppressive therapy [51]. The authors evidenced the presence of antiretroviral resistance mutations above 1% of frequency in five of the eleven patients analyzed (45%). The difference in prevalence found in both studies can be attributed to differences in the ultradeep sequencing method used (MiSeq - Illumina versus 454 Life Science - Roche GS Junior), the regions covered by the study (NFLG versus Gag, Pol and Nef regions) and the associated coverage obtained, as two patients of their cohort only presented mutations under 1%.

Eight of our proviral sequences (8/23, 22%) harbored only minority variants with drug resistance-associated mutations. This finding underscores the use of more sensitive HIV genotyping techniques, since the standard genotypic resistance testing using bulk sequencing of the viral population can only detect viral variants that constitute over 15–20% of the total viral population [65], and likely underestimates the overall prevalence of resistant variants and may impact on the surveillance of HIV resistance and on the clinical management of treated patients.

The clinical significance of drug-resistant minority variants is still uncertain and has been addressed by several studies, due to its potential impact on the response to future treatment schemes. Some studies point out that these resistance variants would be favored by the ARV selective pressure, leading to a substitution of the wild-type virus and consequently to therapeutic failure [21,33,34,35,36,37,38]. However, other studies found no influence of these minority drug-resistance variants on treatment response, highlighting the role of the majority, not the minority variants, in this outcome [39,40,41,42,43,44,45].

Despite the high prevalence of drug resistance mutations (18/23) and of mutations associated with the current ARV therapy of the patients (6/23) in our study, only one patient had detectable viral load 15 months after inclusion in the study. Unfortunately, we failed to PCR-amplify the HIV RT polymerase domain of this patient, and only the E399D mutation was found in RT connection domain of that virus. Most of the drug resistance mutations associated with the current therapy regimens of the patients analyzed were found at low prevalence at the viral population (1.3%–8%), except for the patient harboring TAMs (patient #28; Table 3). Surprisingly, this patient maintained an undetectable viral load 14 months after inclusion in the study, paralleled by an increase in the CD4^+^ T-cell counts (681 cells/mm^3^) compared to those at the time of enrolment (535 cells/mm^3^). The low prevalence observed in most of the mutations found and the high adherence reported by the clinicians of the program may have contributed to the high therapeutic success rate observed in these patients one year after inclusion in the study.

In Brazil, two entry inhibitors/antagonists, enfuvirtide (ENF) and maraviroc (MVC), have been used since 2005 and 2007, respectively, in therapeutic rescue strategies for patients failing previous ARV regimens [97,98]. The resistance mutation to ENF observed in one of our patients (#13; Table 3) emphasizes the importance of including this region at the genotypic resistance analyses, since resistance to ENF is characterized by a low genetic barrier [99,100,101] and can lead to therapeutic failure if used in patients carrying resistance to that drug.

One patient (#11) was found to harbor proviruses with two transmitted drug resistance mutations to protease inhibitors (PI), all of them as minority variants (frequency between 1.9% and 2.8% of the virus population). The treatment history of each patient was considered to define the transmitted resistance. All patients were under NRTI use, so it was not possible to characterize NRTI-associated TDRM. The prevalence of TDR found in this study (1/20; 5%) was in agreement with the moderate TDR prevalence reported by other Brazilian studies, usually ranging from 5 to 10% [102,103,104,105,106,107].

Only three patients studied herein were under PI use in their treatment schemes, and two of those harbored resistance mutations in the protease. While five patients carried integrase inhibitor-related mutations, none used integrase inhibitors, which were only approved as rescue therapy in Brazil at the time of study enrollment. In those cases, mutations were carried from the virus donor to the recipients (transmitted resistance) or arouse in the patient´s virus quasispecies.

With respect to HIV coreceptor tropism, R5 viruses usually predominate during primary HIV infection, whereas the transition to X4 viruses occurs in later stages of HIV disease, being associated with more rapid CD4^+^ T-cell depletion and consequently to AIDS progression [108,109,110,111,112]. Genotypic predictors prove to be highly concordant with phenotypic data and can be reliably used to determine viral tropism with better results in PBMC than in plasma samples [113]. In this study, we used the Geno2pheno algorith with a false positive rate (FPR) cutoff of 10% [54,71,72,73,74]. Prediction of coreceptor usage showed that most individuals (17/22; 77%) presented only R5-tropic viruses, while five individuals presented X4/R5X4-tropic viruses. The prevalence of R5-tropic viruses was similar to the 78% prevalence found by de Azevedo et al. [114] in another Brazilian cohort.

Hypermutated proviral sequences were detected in only one individual (patient #26) through the identification of an excessive G→A change pattern, consistent with APOBEC3G/F signature. Several early stop codons resulted from those nucleotide substitutions were observed along that HIV genome. The evidence of hypermutation as an apolipoprotein B mRNA editing enzyme, catalytic polypeptide-like (APOBEC) action was also highlighted by the Stanford HIV drug resistance database during the analysis of resistance mutations for that virus.

An important limitation of the present study is that the proviral sequences have not been fully evaluated for replication ability, and may be therefore defective or inactive. We only retrieved NFLG sequences from 11 of the 23 patients studied, and no ORF intactness was evaluated for the incomplete genomes. Two of the 11 NFLG sequences had stop codons due to hypermutation or to a frameshift deletion, and are thought to be defective for replication. However, missense mutations that could render the virus defective were not evaluated, and the number of defective proviruses is likely underestimated here.

We are aware that additional studies should be carried out to validate our findings in a larger population. Our strict inclusion criteria did not allow the enrollment of a large cohort for this study. Another limitation was the difficulty of PCR-amplifying the archived proviral genomes from some patients in a setting of undetectable HIV viral load and early chronic infection, where PBMC archived HIV reservoirs are thought to be small. Even with the utilization of diverse strategies, some genomic regions could not be amplified for some patients. Despite we have carried out the PCR amplifications of the virus samples in duplicates to avoid false-positive mutations introduced by PCR errors, we did not quantify the input of proviral copies subjected to the analyses, what could impact the estimates of the variation found in the quasispecies. The subjects under study were in the chronic phase of infection, yet not in advanced stage of the disease (as judged by the abscence of AIDS and the high number of CD4^+^ T-cell counts), and their proviral quaisispecies population was expected to be fully established. In addition, the fact that we achieved rates of 1–2% of frequency for particular DRMs in several patients suggests that we were able to reach such sensitivity rates in our assays.

The analysis of resistance-associated mutations in HIV-positive patients is an important issue when considering the context of broad access to antiretroviral treatment and high rates of therapeutic success, one of the main goals to be achieved worldwide against HIV infection in the coming years. The high rate of resistance-associated mutations found in our cohort, composed of patients with undetectable viral load undergoing HAART as first-line therapy, directs attention to the selection of antiretroviral resistant variants in a context of therapeutic success and early in chroninc infection. These obervations underscore the importance of further studies in order to better correlate the presence of these drug resistanc mutations and response to ART to investigate their potential association with therapeutic failure and to establish effective public policies to decrease their prevalence and transmission.

## Figures and Tables

**Table 1:** Mutations analyzed in the C-terminal domains of reverse transcriptase covering the connection (CN) and RNase H (RH) subdomains and the respective classes of antiretrovirals associated with resistance as described in the literature *.

Subdomain	Mutation	ARV Class Associated with Resistance
CN	E312Q	NRTI
	Y318F/W	NNRTI
	G335D/C (polymorphism)	NRTI
	N348I	NRTI and NNRTI
	A360I/V	NRTI
	V365I	NRTI
	T369I/V	NRTI and NNRTI
	A371V	NRTI
	A376S	NRTI and NNRTI
	E399D/G	NRTI and NNRTI
	A400T (polymorphism)	NRTI
RH	D488E	NRTI
	Q509L	NRTI and NNRTI
	Q547K	NRTI

NRTI, nucleotide/nucleoside reverse transcriptase inhibitors; NNRTI, non-nucleoside reverse transcriptase inhibitors; * References [57,58,59,60,61,62,63,64].

**Table 2:** Demographic and clinical characteristics of the 32 human immunodeficiency virus (HIV)-positive participants of the study.

Characteristic	N
Males (%)	24 (75%)
Age (years) (mean ± SD)	40 ± 12.3
Median baseline CD4^+^ T-cell counts (cells/mm^3^; IQR_50_)	712.5 (606.5–856)
Median baseline CD8^+^ T-cell counts (cells/mm^3^; IQR_50_)	657.5 (529–1047.25)
Median time since HIV diagnosis (years; IQR_50_)	4.7 (3.9–6.5)
Median time from HIV diagnosis to antiretroviral therapy initiation (years; IQR_50_) *	1.2 (0.6–2.8)
Median time of treatment (years; IQR_50_)	3.1 (2.4–3.9)

* Missing in one patient; SD, standard deviation; IQR_50_, interquartile range.

**Table 3:** Distribution of the antiretroviral resistance mutations and major envelope tropism found across the 23 HIV-1 genome sequences analyzed.

Patient	Protease Mutations (Coverage; Frequency)	Reverse Transcriptase Mutations (Coverage; Frequency)	RT Connection Mutations (Coverage; Frequency)	RT RNase H Mutation	Integrase Mutations (Coverage; Frequency)	Envelope Mutations (Coverage; Frequency)	HAART Regimen	Tropism ^1^	Subtype/URF
1	-	M184V (11,979; 2.0%)	-	-	S147G (8945; 1.1%)	-	AZT + 3TC + NVP	100.0% X4/R5X4	B
**2** ^#^	-	-	-	-	R263K (2201; 6.2%)	-	AZT + 3TC + EFV	63.0% X4/R5X4	B
**3**	-	-	-	-	-	-	TDF + 3TC + EFV	97.2% R5	B
**5**	I47V (3589; 10.3%)	-	-	-	-	-	AZT + 3TC + ATV	100% R5	B
8	-	NA	E399D (2701; 99.2%)	-	-	-	TDF + 3TC + EFV	97.1% R5	BC
**11**	D30N (8012; 2.8%) M46I (8639; 1.9%)	M41L (5444; 99.8%)	T369V (9726; 39.6%) E399G (8334; 4.9%)	-	-	-	TDF + 3TC + EFV	96.1% R5	BF
**12**	-	-	-	-	-	-	TDF + 3TC + EFV	84.8% R5	B
**13**	-	E138K (4909; 1.3%)	-	-	-	V38A (2151; 3.0%)	TDF + 3TC + EFV	99.0% X4/R5X4	B
14	-	-	-	NA	R263K * (1776; 11.0%)	-	AZT + 3TC + LPV/r	97.8% R5	B
**15**	-	-	E399G (14,872; 1.5%)	-	-	-	TDF + 3TC + EFV	97.7% R5	B
16	D30N (4574; 49.3%) M46I (4378; 45.6%)	-	-	-	-	-	AZT + 3TC + FPV/r	NA	B
18	-	A62V (4116; 1.0%)	-	-	-	NA	TDF + 3TC + EFV	91.6% R5	B
19	-	NA	A376S (6663; 99.9%)	-	-	-	AZT + 3TC + EFV	99.6% R5	B
20	-	-	-	NA	NA	NA	TDF + 3TC + EFV	100.0% R5	B
**21**	-	L210W (12,583; 100.0%)	-	-	-	-	TDF + EFV + FTC	99.3% R5	B
22	-	NA	A376S * (1082; 94.9%)	-	-	-	TDF + 3TC + EFV	100.0% R5	B
**23**	-	-	-	-	T97A (4113; 2.1%)	-	TDF + 3TC + EFV	96.0% R5	B
26	-	NA	NA	NA	NA	-	TDF + 3TC + EFV	100.0% X4/R5X4	B
27	-	NA	-	-	-	-	TDF + 3TC + EFV	99.9% R5	B
28	-	M41L (5791; 99.5%) D67N (6465; 72.9%) K70R (6475; 77.2%) T215Y (9219; 38.7%)	E399D (2051; 99.9%)	NA	NA	-	TDF + 3TC + EFV	99.1% R5	BF
29	-	-	E399D (5209; 100.0%)	-	T66I (7444; 31.0%)	NA	TDF + 3TC + EFV	99.7% X4/R5X4	B
**31**	-	M184V (2708; 1.3%)	-	-	-	-	TDF + 3TC + EFV	100.0% R5	B
**32**	-	V179D (7146; 99.3%) M184V (7127; 8.0%)	-	-	-	-	TDF + 3TC + EFV	100.0% R5	BF

^#^ The near full-length genomes are in bold; NA, not available; -, no mutations found; *, only partial sequence available; ^1^ X4/R5X4: CXC chemokine receptor 4 (CXCR4) and/or CXCR4/CC chemokine receptor 5 (CCR5) tropism profile; R5: CCR5 tropism profile.

## Supplementary Materials

The HIV-1 consensus sequences were deposited at the GenBank nucleotide database under the accession numbers MG571979-MG572011. The raw NGS reads were submitted to the Sequence Read Archive (SRA) under the numbers SRR6324863-SRR6324885. The following are available online at www.mdpi.com/1999-4915/9/12/392/s1.

## References

[1] The Joint United Nations Programme on HIV/AIDS (UNAIDS) (2017). UNAIDS Data 2017.

[2] The Joint United Nations Programme on HIV/AIDS (UNAIDS) (2017). Ending AIDS: Progress Towards the 90-90-90 Targets.

[3] Brazilian Ministry of Health (2017). Clinical Protocol and Therapeutic Guidelines for the Management of HIV Infection in Adults.

[4] Brazilian Ministry of Health (2013). Clinical Protocol and Therapeutic Guidelines for Management of HIV Infection in Adults.

[5] Roberts J.D., Bebenek K., Kunkel T.A. (1988). The accuracy of reverse transcriptase from HIV-1. Science.

[6] Quan Y., Xu H., Wainberg M.A. (2014). Defective HIV-1 quasispecies in the form of multiply drug-resistant proviral DNA within cells can be rescued by superinfection with different subtype variants of HIV-1 and by HIV-2 and SIV. J. Antimicrob. Chemother..

[7] Wain-Hobson S. (1993). The fastest genome evolution ever described: HIV variation in situ. Curr. Opin. Genet. Dev..

[8] Overbaugh J., Bangham C.R. (2001). Selection forces and constraints on retroviral sequence variation. Science.

[9] Atlas A., Granath F., Lindstrom A., Lidman K., Lindback S., Alaeus A. (2005). Impact of HIV type 1 genetic subtype on the outcome of antiretroviral therapy. AIDS Res. Hum. Retrovir..

[10] Tang M.W., Shafer R.W. (2012). HIV-1 antiretroviral resistance: Scientific principles and clinical applications. Drugs.

[11] Bennett D.E., Camacho R.J., Otelea D., Kuritzkes D.R., Fleury H., Kiuchi M., Heneine W., Kantor R., Jordan M.R., Schapiro J.M. (2009). Drug resistance mutations for surveillance of transmitted HIV-1 drug-resistance: 2009 update. PLoS ONE.

[12] Shafer R.W., Rhee S.Y., Pillay D., Miller V., Sandstrom P., Schapiro J.M., Kuritzkes D.R., Bennett D. (2007). HIV-1 protease and reverse transcriptase mutations for drug resistance surveillance. AIDS.

[13] Castor D., Low A., Evering T., Karmon S., Davis B., Figueroa A., LaMar M., Garmon D., Mehandru S., Markowitz M. (2012). Transmitted drug resistance and phylogenetic relationships among acute and early HIV-1-infected individuals in New York City. J. Acquir. Immune Defic. Syndr..

[14] Olson A., Bannert N., Sonnerborg A., de Mendoza C., Price M., Zangerle R., Chaix M.L., Prins M., Kran A.B., Gill J. (2017). Temporal trends of transmitted HIV drug resistance in a multinational seroconversion cohort. AIDS.

[15] Reynolds S.J., Ssempijja V., Galiwango R., Ndyanabo A., Nakigozi G., Lyagoba F., Nazziwa J., Redd A., Lamers S.L., Gray R. (2017). Low rates of transmitted drug resistance among newly identified HIV-1 seroconverters in Rural Rakai, Uganda. AIDS Res. Hum. Retrovir..

[16] Zhang F., Liu L., Sun M., Sun J., Lu H. (2017). An analysis of drug resistance among people living with HIV/AIDS in Shanghai, China. PLoS ONE.

[17] Vrancken B., Trovao N.S., Baele G., van Wijngaerden E., Vandamme A.M., van Laethem K., Lemey P. (2016). Quantifying next generation sequencing sample pre-processing bias in HIV-1 complete genome sequencing. Viruses.

[18] Grant R.M., Kuritzkes D.R., Johnson V.A., Mellors J.W., Sullivan J.L., Swanstrom R., D’Aquila R.T., van Gorder M., Holodniy M., Lloyd R.M. (2003). Accuracy of the TRUGENE HIV-1 genotyping kit. J. Clin. Microbiol..

[19] Halvas E.K., Aldrovandi G.M., Balfe P., Beck I.A., Boltz V.F., Coffin J.M., Frenkel L.M., Hazelwood J.D., Johnson V.A., Kearney M. (2006). Blinded, multicenter comparison of methods to detect a drug-resistant mutant of human immunodeficiency virus type 1 at low frequency. J. Clin. Microbiol..

[20] Larder B.A., Kohli A., Kellam P., Kemp S.D., Kronick M., Henfrey R.D. (1993). Quantitative detection of HIV-1 drug resistance mutations by automated DNA sequencing. Nature.

[21] Bellecave P., Recordon-Pinson P., Papuchon J., Vandenhende M.A., Reigadas S., Tauzin B., Fleury H. (2014). Detection of low-frequency HIV type 1 reverse transcriptase drug resistance mutations by ultradeep sequencing in naive HIV type 1-infected individuals. AIDS Res. Hum. Retrovir..

[22] Dudley D.M., Chin E.N., Bimber B.N., Sanabani S.S., Tarosso L.F., Costa P.R., Sauer M.M., Kallas E.G., O’Connor D.H. (2012). Low-cost ultra-wide genotyping using Roche/454 pyrosequencing for surveillance of HIV drug resistance. PLoS ONE.

[23] Gonzalez S., Tully D.C., Gondwe C., Wood C. (2013). Low-abundance resistant mutations in HIV-1 subtype C antiretroviral therapy-naive individuals as revealed by pyrosequencing. Curr. HIV Res..

[24] Ji H., Li Y., Graham M., Liang B.B., Pilon R., Tyson S., Peters G., Tyler S., Merks H., Bertagnolio S. (2011). Next-generation sequencing of dried blood spot specimens: A novel approach to HIV drug-resistance surveillance. Antivir. Ther..

[25] Simen B.B., Simons J.F., Hullsiek K.H., Novak R.M., Macarthur R.D., Baxter J.D., Huang C., Lubeski C., Turenchalk G.S., Braverman M.S. (2009). Terry Beirn Community Programs for Clinical Research on, A. Low-abundance drug-resistant viral variants in chronically HIV-infected, antiretroviral treatment-naive patients significantly impact treatment outcomes. J. Infect. Dis..

[26] Liu L., Li Y., Li S., Hu N., He Y., Pong R., Lin D., Lu L., Law M. (2012). Comparison of next-generation sequencing systems. J. Biomed. Biotechnol..

[27] Cane P.A. (2009). New developments in HIV drug resistance. J. Antimicrob. Chemother..

[28] Coffin J.M. (1995). HIV population dynamics in vivo: Implications for genetic variation, pathogenesis, and therapy. Science.

[29] Metzner K.J., Rauch P., Walter H., Boesecke C., Zollner B., Jessen H., Schewe K., Fenske S., Gellermann H., Stellbrink H.J. (2005). Detection of minor populations of drug-resistant HIV-1 in acute seroconverters. AIDS.

[30] Ghosn J., Pellegrin I., Goujard C., Deveau C., Viard J.P., Galimand J., Harzic M., Tamalet C., Meyer L., Rouzioux C. (2006). HIV-1 resistant strains acquired at the time of primary infection massively fuel the cellular reservoir and persist for lengthy periods of time. AIDS.

[31] Booth C.L., Geretti A.M. (2007). Prevalence and determinants of transmitted antiretroviral drug resistance in HIV-1 infection. J. Antimicrob. Chemother..

[32] Pingen M., Nijhuis M., de Bruijn J.A., Boucher C.A., Wensing A.M. (2011). Evolutionary pathways of transmitted drug-resistant HIV-1. J. Antimicrob. Chemother..

[33] Kyeyune F., Gibson R.M., Nankya I., Venner C., Metha S., Akao J., Ndashimye E., Kityo C.M., Salata R.A., Mugyenyi P. (2016). Low-Frequency drug resistance in HIV-infected ugandans on antiretroviral treatment is associated with regimen failure. Antimicrob. Agents Chemother..

[34] Lataillade M., Chiarella J., Yang R., Schnittman S., Wirtz V., Uy J., Seekins D., Krystal M., Mancini M., McGrath D. (2010). Prevalence and clinical significance of HIV drug resistance mutations by ultra-deep sequencing in antiretroviral-naive subjects in the CASTLE study. PLoS ONE.

[35] Vandenhende M.A., Bellecave P., Recordon-Pinson P., Reigadas S., Bidet Y., Bruyand M., Bonnet F., Lazaro E., Neau D., Fleury H. (2014). Prevalence and evolution of low frequency HIV drug resistance mutations detected by ultra deep sequencing in patients experiencing first line antiretroviral therapy failure. PLoS ONE.

[36] Nishizawa M., Matsuda M., Hattori J., Shiino T., Matano T., Heneine W., Johnson J.A., Sugiura W. (2015). Longitudinal detection and persistence of minority drug-resistant populations and their effect on salvage therapy. PLoS ONE.

[37] Johnson J.A., Li J.F., Wei X., Lipscomb J., Irlbeck D., Craig C., Smith A., Bennett D.E., Monsour M., Sandstrom P. (2008). Minority HIV-1 drug resistance mutations are present in antiretroviral treatment-naive populations and associate with reduced treatment efficacy. PLoS Med..

[38] Pingen M., van der Ende M.E., Wensing A.M., El Barzouhi A., Simen B.B., Schutten M., Boucher C.A. (2013). Deep sequencing does not reveal additional transmitted mutations in patients diagnosed with HIV-1 variants with single nucleoside reverse transcriptase inhibitor resistance mutations. HIV Med..

[39] Peuchant O., Thiebaut R., Capdepont S., Lavignolle-Aurillac V., Neau D., Morlat P., Dabis F., Fleury H., Masquelier B., Cohort A.C.A. (2008). Transmission of HIV-1 minority-resistant variants and response to first-line antiretroviral therapy. AIDS.

[40] Boltz V.F., Ambrose Z., Kearney M.F., Shao W., Kewalramani V.N., Maldarelli F., Mellors J.W., Coffin J.M. (2012). Ultrasensitive allele-specific PCR reveals rare preexisting drug-resistant variants and a large replicating virus population in macaques infected with a simian immunodeficiency virus containing human immunodeficiency virus reverse transcriptase. J. Virol..

[41] Metzner K.J., Rauch P., von Wyl V., Leemann C., Grube C., Kuster H., Boni J., Weber R., Gunthard H.F. (2010). Efficient suppression of minority drug-resistant HIV type 1 (HIV-1) variants present at primary HIV-1 infection by ritonavir-boosted protease inhibitor-containing antiretroviral therapy. J. Infect. Dis..

[42] Gianella S., Delport W., Pacold M.E., Young J.A., Choi J.Y., Little S.J., Richman D.D., Kosakovsky Pond S.L., Smith D.M. (2011). Detection of minority resistance during early HIV-1 infection: Natural variation and spurious detection rather than transmission and evolution of multiple viral variants. J. Virol..

[43] Stekler J.D., Ellis G.M., Carlsson J., Eilers B., Holte S., Maenza J., Stevens C.E., Collier A.C., Frenkel L.M. (2011). Prevalence and impact of minority variant drug resistance mutations in primary HIV-1 infection. PLoS ONE.

[44] Lataillade M., Chiarella J., Yang R., DeGrosky M., Uy J., Seekins D., Simen B., St John E., Moreno E., Kozal M. (2012). Virologic failures on initial boosted-PI regimen infrequently possess low-level variants with major PI resistance mutations by ultra-deep sequencing. PLoS ONE.

[45] Charpentier C., Lee G.Q., Rodriguez C., Visseaux B., Storto A., Fagard C., Molina J.M., Katlama C., Yazdanpanah Y., Harrigan P.R. (2015). Highly frequent HIV-1 minority resistant variants at baseline of the ANRS 139 TRIO trial had a limited impact on virological response. J. Antimicrob. Chemother..

[46] Hare C.B., Mellors J., Krambrink A., Su Z., Skiest D., Margolis D.M., Patel S.S., Barnas D., Frenkel L., Coombs R.W. (2008). Detection of nonnucleoside reverse-transcriptase inhibitor-resistant HIV-1 after discontinuation of virologically suppressive antiretroviral therapy. Clin. Infect. Dis..

[47] Le T., Chiarella J., Simen B.B., Hanczaruk B., Egholm M., Landry M.L., Dieckhaus K., Rosen M.I., Kozal M.J. (2009). Low-abundance HIV drug-resistant viral variants in treatment-experienced persons correlate with historical antiretroviral use. PLoS ONE.

[48] Li J.Z., Paredes R., Ribaudo H.J., Svarovskaia E.S., Metzner K.J., Kozal M.J., Hullsiek K.H., Balduin M., Jakobsen M.R., Geretti A.M. (2011). Low-frequency HIV-1 drug resistance mutations and risk of NNRTI-based antiretroviral treatment failure: A systematic review and pooled analysis. JAMA.

[49] Cozzi-Lepri A., Noguera-Julian M., di Giallonardo F., Schuurman R., Daumer M., Aitken S., Ceccherini-Silberstein F., D’Arminio Monforte A., Geretti A.M., Booth C.L. (2015). Low-frequency drug-resistant HIV-1 and risk of virological failure to first-line NNRTI-based ART: A multicohort European case-control study using centralized ultrasensitive 454 pyrosequencing. J. Antimicrob. Chemother..

[50] Paredes R., Lalama C.M., Ribaudo H.J., Schackman B.R., Shikuma C., Giguel F., Meyer W.A., Johnson V.A., Fiscus S.A., D’Aquila R.T. (2010). Pre-existing minority drug-resistant HIV-1 variants, adherence, and risk of antiretroviral treatment failure. J. Infect. Dis..

[51] Papuchon J., Pinson P., Lazaro E., Reigadas S., Guidicelli G., Taupin J.L., Neau D., Fleury H., The Provir/Latitude 45 Project (2013). Resistance mutations and CTL epitopes in archived HIV-1 DNA of patients on antiviral treatment: Toward a new concept of vaccine. PLoS ONE.

[52] CDC (2014). Revised surveillance case definition for HIV infection—United States, 2014. Morb. Mortal. Wkly. Rep..

[53] Sanabani S., Neto W.K., de Sa Filho D.J., Diaz R.S., Munerato P., Janini L.M., Sabino E.C. (2006). Full-length genome analysis of human immunodeficiency virus type 1 subtype C in Brazil. AIDS Res. Hum. Retrovir..

[54] Ode H., Matsuda M., Matsuoka K., Hachiya A., Hattori J., Kito Y., Yokomaku Y., Iwatani Y., Sugiura W. (2015). Quasispecies Analyses of the HIV-1 Near-full-length Genome With Illumina MiSeq. Front. Microbiol..

[55] Dudley D.M., Bailey A.L., Mehta S.H., Hughes A.L., Kirk G.D., Westergaard R.P., O’Connor D.H. (2014). Cross-clade simultaneous HIV drug resistance genotyping for reverse transcriptase, protease, and integrase inhibitor mutations by Illumina MiSeq. Retrovirology.

[56] Wensing A.M., Calvez V., Gunthard H.F., Johnson V.A., Paredes R., Pillay D., Shafer R.W., Richman D.D. (2015). 2015 Update of the Drug Resistance Mutations in HIV-1. Top. Antivir. Med..

[57] Brehm J.H., Koontz D., Meteer J.D., Pathak V., Sluis-Cremer N., Mellors J.W. (2007). Selection of mutations in the connection and RNase H domains of human immunodeficiency virus type 1 reverse transcriptase that increase resistance to 3′-azido-3′-dideoxythymidine. J. Virol..

[58] Dau B., Ayers D., Singer J., Harrigan P.R., Brown S., Kyriakides T., Cameron D.W., Angus B., Holodniy M. (2010). Connection domain mutations in treatment-experienced patients in the OPTIMA trial. J. Acquir. Immune Defic. Syndr..

[59] Delviks-Frankenberry K.A., Nikolenko G.N., Maldarelli F., Hase S., Takebe Y., Pathak V.K. (2009). Subtype-specific differences in the human immunodeficiency virus type 1 reverse transcriptase connection subdomain of CRF01_AE are associated with higher levels of resistance to 3′-azido-3′-deoxythymidine. J. Virol..

[60] Delviks-Frankenberry K.A., Nikolenko G.N., Pathak V.K. (2010). The “Connection” Between HIV Drug Resistance and RNase H. Viruses.

[61] Gupta S., Vingerhoets J., Fransen S., Tambuyzer L., Azijn H., Frantzell A., Paredes R., Coakley E., Nijs S., Clotet B. (2011). Connection domain mutations in HIV-1 reverse transcriptase do not impact etravirine susceptibility and virologic responses to etravirine-containing regimens. Antimicrob. Agents Chemother..

[62] Lengruber R.B., Delviks-Frankenberry K.A., Nikolenko G.N., Baumann J., Santos A.F., Pathak V.K., Soares M.A. (2011). Phenotypic characterization of drug resistance-associated mutations in HIV-1 RT connection and RNase H domains and their correlation with thymidine analogue mutations. J. Antimicrob. Chemother..

[63] Paredes R., Puertas M.C., Bannister W., Kisic M., Cozzi-Lepri A., Pou C., Bellido R., Betancor G., Bogner J., Gargalianos P. (2011). A376S in the connection subdomain of HIV-1 reverse transcriptase confers increased risk of virological failure to nevirapine therapy. J. Infect. Dis..

[64] Santos A.F., Lengruber R.B., Soares E.A., Jere A., Sprinz E., Martinez A.M., Silveira J., Sion F.S., Pathak V.K., Soares M.A. (2008). Conservation patterns of HIV-1 RT connection and RNase H domains: Identification of new mutations in NRTI-treated patients. PLoS ONE.

[65] Eshleman S.H., Hackett J., Swanson P., Cunningham S.P., Drews B., Brennan C., Devare S.G., Zekeng L., Kaptue L., Marlowe N. (2004). Performance of the Celera Diagnostics ViroSeq HIV-1 Genotyping System for sequence-based analysis of diverse human immunodeficiency virus type 1 strains. J. Clin. Microbiol..

[66] Guindon S., Dufayard J.F., Lefort V., Anisimova M., Hordijk W., Gascuel O. (2010). New algorithms and methods to estimate maximum-likelihood phylogenies: Assessing the performance of PhyML 3.0. Syst. Biol..

[67] Keane T.M., Creevey C.J., Pentony M.M., Naughton T.J., McLnerney J.O. (2006). Assessment of methods for amino acid matrix selection and their use on empirical data shows that ad hoc assumptions for choice of matrix are not justified. BMC Evol. Biol..

[68] Lole K.S., Bollinger R.C., Paranjape R.S., Gadkari D., Kulkarni S.S., Novak N.G., Ingersoll R., Sheppard H.W., Ray S.C. (1999). Full-length human immunodeficiency virus type 1 genomes from subtype C-infected seroconverters in India, with evidence of intersubtype recombination. J. Virol..

[69] Jeanne N., Saliou A., Carcenac R., Lefebvre C., Dubois M., Cazabat M., Nicot F., Loiseau C., Raymond S., Izopet J. (2015). Position-specific automated processing of V3 env ultra-deep pyrosequencing data for predicting HIV-1 tropism. Sci. Rep..

[70] Lengauer T., Sander O., Sierra S., Thielen A., Kaiser R. (2007). Bioinformatics prediction of HIV coreceptor usage. Nat. Biotechnol..

[71] Delgado E., Fernandez-Garcia A., Vega Y., Cuevas T., Pinilla M., Garcia V., Sanchez M., Gonzalez M., Sanchez A.M., Thomson M.M. (2012). Evaluation of genotypic tropism prediction tests compared with in vitro co-receptor usage in HIV-1 primary isolates of diverse subtypes. J. Antimicrob. Chemother..

[72] Gibson R.M., Meyer A.M., Winner D., Archer J., Feyertag F., Ruiz-Mateos E., Leal M., Robertson D.L., Schmotzer C.L., Quinones-Mateu M.E. (2014). Sensitive deep-sequencing-based HIV-1 genotyping assay to simultaneously determine susceptibility to protease, reverse transcriptase, integrase, and maturation inhibitors, as well as HIV-1 coreceptor tropism. Antimicrob. Agents Chemother..

[73] Raymond S., Delobel P., Mavigner M., Ferradini L., Cazabat M., Souyris C., Sandres-Saune K., Pasquier C., Marchou B., Massip P. (2010). Prediction of HIV type 1 subtype C tropism by genotypic algorithms built from subtype B viruses. J. Acquir. Immune Defic. Syndr..

[74] Vandekerckhove L.P., Wensing A.M., Kaiser R., Brun-Vezinet F., Clotet B., de Luca A., Dressler S., Garcia F., Geretti A.M., Klimkait T. (2011). European Consensus Group on clinical management of tropism, t., European guidelines on the clinical management of HIV-1 tropism testing. Lancet Infect. Dis..

[75] Kaleebu P., French N., Mahe C., Yirrell D., Watera C., Lyagoba F., Nakiyingi J., Rutebemberwa A., Morgan D., Weber J. (2002). Effect of human immunodeficiency virus (HIV) type 1 envelope subtypes A and D on disease progression in a large cohort of HIV-1-positive persons in Uganda. J. Infect. Dis..

[76] Archary D., Gordon M.L., Green T.N., Coovadia H.M., Goulder P.J., Ndung’u T. (2010). HIV-1 subtype C envelope characteristics associated with divergent rates of chronic disease progression. Retrovirology.

[77] Casado C., Colombo S., Rauch A., Martinez R., Gunthard H.F., Garcia S., Rodriguez C., del Romero J., Telenti A., Lopez-Galindez C. (2010). Host and viral genetic correlates of clinical definitions of HIV-1 disease progression. PLoS ONE.

[78] McLaren P.J., Carrington M. (2015). The impact of host genetic variation on infection with HIV-1. Nat. Immunol..

[79] Leite T.C., Campos D.P., Coelho A.B., Teixeira S.L., Veloso V., Morgado M.G., Guimaraes M.L. (2017). Impact of HIV-1 subtypes on AIDS progression in a Brazilian cohort. AIDS Res. Hum. Retrovir..

[80] Osmanov S., Pattou C., Walker N., Schwardlander B., Esparza J., WHO-UNAIDS Network for HIV Isolation and Characterization (2002). Estimated global distribution and regional spread of HIV-1 genetic subtypes in the year 2000. J. Acquir. Immune Defic. Syndr..

[81] Hemelaar J., Gouws E., Ghys P.D., Osmanov S., WHO-UNAIDS Network for HIV Isolation and Characterisation (2011). Global trends in molecular epidemiology of HIV-1 during 2000–2007. AIDS.

[82] Junqueira D.M., Almeida S.E. (2016). HIV-1 subtype B: Traces of a pandemic. Virology.

[83] Santos A.F., Schrago C.G., Martinez A.M., Mendoza-Sassi R., Silveira J., Sousa T.M., Lengruber R.B., Soares E.A., Sprinz E., Soares M.A. (2007). Epidemiologic and evolutionary trends of HIV-1 CRF31_BC-related strains in southern Brazil. J. Acquir. Immune Defic. Syndr..

[84] Passaes C.P., Guimaraes M.L., Bello G., Morgado M.G. (2009). Near full-length genome characterization of HIV type 1 unique BC recombinant forms from Southern Brazil. AIDS Res. Hum. Retrovir..

[85] Almeida S.E., de Medeiros R.M., Junqueira D.M., Graf T., Passaes C.P., Bello G., Morgado M.G., Guimarães M.L. (2012). Temporal dynamics of HIV-1 circulating subtypes in distinct exposure categories in southern Brazil. Virol. J..

[86] Cardoso L.P., Queiroz B.B., Stefani M.M. (2009). HIV-1 pol phylogenetic diversity and antiretroviral resistance mutations in treatment naive patients from Central West Brazil. J. Clin. Virol..

[87] De Medeiros R.M., Junqueira D.M., Matte M.C., Barcellos N.T., Chies J.A., Matos Almeida S.E. (2011). Co-circulation HIV-1 subtypes B, C, and CRF31_BC in a drug-naive population from Southernmost Brazil: Analysis of primary resistance mutations. J. Med. Virol..

[88] Graf T., Pinto A.R. (2013). The increasing prevalence of HIV-1 subtype C in Southern Brazil and its dispersion through the continent. Virology.

[89] Sanabani S.S., Pessoa R., Soares de Oliveira A.C., Martinez V.P., Giret M.T., de Menezes Succi R.C., Carvalho K., Tomiyama C.S., Nixon D.F., Sabino E.C. (2013). Variability of HIV-1 genomes among children and adolescents from Sao Paulo, Brazil. PLoS ONE.

[90] Machado L.F., Ishak M.O., Vallinoto A.C., Lemos J.A., Azevedo V.N., Moreira M.R., Souza M.I., Fernandes L.M., Souza L.L., Ishak R. (2009). Molecular epidemiology of HIV type 1 in northern Brazil: Identification of subtypes C and D and the introduction of CRF02_AG in the Amazon region of Brazil. AIDS Res. Hum. Retrovir..

[91] Pessoa R., Loureiro P., Esther Lopes M., Carneiro-Proietti A.B., Sabino E.C., Busch M.P., Sanabani S.S. (2016). Ultra-Deep Sequencing of HIV-1 near Full-Length and Partial Proviral Genomes Reveals High Genetic Diversity among Brazilian Blood Donors. PLoS ONE.

[92] Velasco-de-Castro C.A., Grinsztejn B., Veloso V.G., Bastos F.I., Pilotto J.H., Fernandes N., Morgado M.G. (2014). HIV-1 diversity and drug resistance mutations among people seeking HIV diagnosis in voluntary counseling and testing sites in Rio de Janeiro, Brazil. PLoS ONE.

[93] Noe A., Plum J., Verhofstede C. (2005). The latent HIV-1 reservoir in patients undergoing HAART: An archive of pre-HAART drug resistance. J. Antimicrob. Chemother..

[94] Bon I., Alessandrini F., Borderi M., Gorini R., Re M.C. (2007). Analysis of HIV-1 drug-resistant variants in plasma and peripheral blood mononuclear cells from untreated individuals: Implications for clinical management. New Microbiol..

[95] Wirden M., Soulie C., Valantin M.A., Fourati S., Simon A., Lambert-Niclot S., Bonmarchand M., Clavel-Osorio C., Marcelin A.G., Katlama C. (2011). Historical HIV-RNA resistance test results are more informative than proviral DNA genotyping in cases of suppressed or residual viraemia. J. Antimicrob. Chemother..

[96] Jakobsen M.R., Tolstrup M., Sogaard O.S., Jorgensen L.B., Gorry P.R., Laursen A., Ostergaard L. (2010). Transmission of HIV-1 drug-resistant variants: Prevalence and effect on treatment outcome. Clin. Infect. Dis..

[97] Alencar C.S., Nishiya A.S., Ferreira S., Giret M.T., Diaz R.S., Sabino E.C. (2010). Evaluation of primary resistance to HIV entry inhibitors among brazilian patients failing reverse transcriptase/protease inhibitors treatment reveal high prevalence of maraviroc resistance-related mutations. AIDS Res. Hum. Retrovir..

[98] Araujo L.A., Junqueira D.M., de Medeiros R.M., Matte M.C., Almeida S.E. (2012). Naturally occurring resistance mutations to HIV-1 entry inhibitors in subtypes B, C, and CRF31_BC. J. Clin. Virol..

[99] Sista P.R., Melby T., Davison D., Jin L., Mosier S., Mink M., Nelson E.L., DeMasi R., Cammack N., Salgo M.P. (2004). Characterization of determinants of genotypic and phenotypic resistance to enfuvirtide in baseline and on-treatment HIV-1 isolates. AIDS.

[100] Poveda E., Rodes B., Labernardiere J.L., Benito J.M., Toro C., Gonzalez-Lahoz J., Faudon J.L., Clavel F., Schapiro J., Soriano V. (2004). Evolution of genotypic and phenotypic resistance to Enfuvirtide in HIV-infected patients experiencing prolonged virologic failure. J. Med. Virol..

[101] Poveda E., Rodes B., Lebel-Binay S., Faudon J.L., Jimenez V., Soriano V. (2005). Dynamics of enfuvirtide resistance in HIV-infected patients during and after long-term enfuvirtide salvage therapy. J. Clin. Virol..

[102] Brindeiro R.M., Diaz R.S., Sabino E.C., Morgado M.G., Pires I.L., Brigido L., Dantas M.C., Barreira D., Teixeira P.R., Tanuri A. (2003). Brazilian Network for Drug Resistance, S. Brazilian Network for HIV Drug Resistance Surveillance (HIV-BResNet): A survey of chronically infected individuals. AIDS.

[103] Gonsalez C.R., Alcalde R., Nishiya A., Barreto C.C., Silva F.E., de Almeida A., Mendonca M., Ferreira F., Fernandes S.S., Casseb J. (2007). Drug resistance among chronic HIV-1-infected patients naive for use of anti-retroviral therapy in Sao Paulo city. Virus Res..

[104] Ferreira A.S., Cardoso L.P., Stefani M.M. (2011). Moderate prevalence of transmitted drug resistance and high HIV-1 genetic diversity in patients from Mato Grosso State, Central Western Brazil. J. Med. Virol..

[105] Inocencio L.A., Pereira A.A., Sucupira M.C., Fernandez J.C., Jorge C.P., Souza D.F., Fink H.T., Diaz R.S., Becker I.M., Suffert T.A. (2009). Brazilian Network for HIV Drug Resistance Surveillance: A survey of individuals recently diagnosed with HIV. J. Int. AIDS Soc..

[106] Pilotto J.H., Grinsztejn B., Veloso V.G., Velasque L.S., Friedman R.K., Moreira R.I., Rodrigues-Pedro A., Oliveira S.M., Currier J.S., Morgado M.G. (2013). Moderate prevalence of transmitted drug resistance mutations among antiretroviral-naive HIV-infected pregnant women in Rio de Janeiro, Brazil. AIDS Res. Hum. Retrovir..

[107] Sprinz E., Netto E.M., Patelli M., Lima J.S., Furtado J.J., da Eira M., Zajdenverg R., Madruga J.V., Lewi D.S., Machado A.A. (2009). Primary antiretroviral drug resistance among HIV type 1-infected individuals in Brazil. AIDS Res. Hum. Retrovir..

[108] Hunt P.W., Harrigan P.R., Huang W., Bates M., Williamson D.W., McCune J.M., Price R.W., Spudich S.S., Lampiris H., Hoh R. (2006). Prevalence of CXCR4 tropism among antiretroviral-treated HIV-1-infected patients with detectable viremia. J. Infect. Dis..

[109] Koot M., Keet I.P., Vos A.H., de Goede R.E., Roos M.T., Coutinho R.A., Miedema F., Schellekens P.T., Tersmette M. (1993). Prognostic value of HIV-1 syncytium-inducing phenotype for rate of CD4^+^ cell depletion and progression to AIDS. Ann. Intern. Med..

[110] Moyle G.J., Wildfire A., Mandalia S., Mayer H., Goodrich J., Whitcomb J., Gazzard B.G. (2005). Epidemiology and predictive factors for chemokine receptor use in HIV-1 infection. J. Infect. Dis..

[111] Scarlatti G., Tresoldi E., Bjorndal A., Fredriksson R., Colognesi C., Deng H.K., Malnati M.S., Plebani A., Siccardi A.G., Littman D.R. (1997). In vivo evolution of HIV-1 co-receptor usage and sensitivity to chemokine-mediated suppression. Nat. Med..

[112] Schuitemaker H., Koot M., Kootstra N.A., Dercksen M.W., de Goede R.E., van Steenwijk R.P., Lange J.M., Schattenkerk J.K., Miedema F., Tersmette M. (1992). Biological phenotype of human immunodeficiency virus type 1 clones at different stages of infection: Progression of disease is associated with a shift from monocytotropic to T-cell-tropic virus population. J. Virol..

[113] Skrabal K., Low A.J., Dong W., Sing T., Cheung P.K., Mammano F., Harrigan P.R. (2007). Determining human immunodeficiency virus coreceptor use in a clinical setting: Degree of correlation between two phenotypic assays and a bioinformatic model. J. Clin. Microbiol..

[114] de Azevedo S.S.D., Caetano D.G., Cortes F.H., Teixeira S.L.M., Dos Santos Silva K., Hoagland B., Grinsztejn B., Veloso V.G., Morgado M.G., Bello G. (2017). Highly divergent patterns of genetic diversity and evolution in proviral quasispecies from HIV controllers. Retrovirology.

