# Supplementary material for: Characterization of HIV-1 Near Full-Length Proviral Genome Quasispecies from Patients with Undetectable Viral Load Undergoing First-Line HAART Therapy
Source: Viruses. 2017 Dec 19;9(12):392. doi: 10.3390/v9120392 (PMC5744166; doi:10.3390/v9120392)
Supplement: Supplementary file 1 [file viruses-09-00392-s001.pdf]

Table S1. Description of the primers used in this study for HIV-1 NFLG amplification by PCR (1st round) and nested PCR (2nd round)

Gene	Primer name	Round	Orientation	Sequence	Localization*
gag-pol	*SC-AOS*	1st	Forward	5′AGA TCT GAG CCT GGG AGC TCT CTG G 3′	521 – 554
	SC-AOA	1st	Reverse	5′TTT ATG GCA AAT ACT GGA GTA TTG TAT GGA 3′	2711 – 2740
	SC-ANS	2nd	Forward	5′GCT TCA AGT AGT GTG TGC CCG TCT G 3′	546 – 570
	SC-ANA	2nd	Reverse	5′CTT TTG GGC CAT CCA TTC CTG 3′	2590 – 2610
pol	*SC-BOS*	1st	Forward	5′GCT AAT TTT TTA GGG AAG ATC TGG CCT T 3′	2080 – 2107
	IN-B02R	1st	Reverse	5′AAA TCA CTA GCC ATT GCT CTC CA 3′	4284 – 4306
	SC-BNS	2nd	Forward	5′AGC CCC ACC AGA AGA GAG CTT 3′	2157 – 2177
	IN-B01R	2nd	Reverse	5′CCA CTC AGG AAT CCA GGT GG 3′	3772 – 3791
pol-vif	*IN-B2OS*	1st	Forward	5′CTC ARG ACT TYT GGG AAG TTC 3′	2800 – 2820
	SC-BOA	1st	Reverse	5′TCT CCT GTA TGC AGA CCC CAA TAT GT 3′	5242 – 5267
	IN-B01S	2nd	Forward	5′GAT GGG TTA TGA ACT CCA TCC TG 3′	3772 – 3791
	C-BNA	2nd	Reverse	5′CCC TAG TGG GAT GTG TAC TTC TGA ACT TA 3′	5192 – 5220
pol, vif, vpr, tat, rev, vpu, env	*SC-COS*	1st	Forward	5′TAC AGT GCA GGG GAA AGA ATA ATA GAC ATA ATA 3′	4809 – 4841
	SC-COA	1st	Reverse	5′TGT CTG GCC TGT ACC GTC AGC G 3′	7831 – 7852
	SC-CNS	2nd	Forward	5′CAA AAT TTT CGG GTT TAT TAC AGG GAC A 3′	4890 – 4917
	SC-CNA	2nd	Reverse	5′GCT TCC TGC TGC TCC CAA GAA CC 3′	7786 – 7808
env, tat, rev, nef	*SC-DOS*	1st	Forward	5′TTG AAC CAY TAG GAG TAG CAC CCA C 3′	7696 – 7720
	SC-DOA	1st	Reverse	5′AGA GAG ACC CAG TAC AGG CAA AAG C 3′	9523 – 9548
	SC-DNS	2nd	Forward	5′ACC AAG GCA AAG AGA AGA GTG GTG 3′	7719 – 7742
	SC-DNA	2nd	Reverse	5′GTA CAG GCA AAA AGC AGC TGC TTA TAT G 3′	9510 – 9537

	1ODEAF	1st	Forward	ATCTCTAGCAGTGGCGCCCGAACAG	625-649
	1ODEAR	1st	Reverse	GCTATTAAGTCTTTTGATGGGTCATA	3529-3504
gag-rt	2ODEAF	2nd	Forward	CTCTCTCGACGCAGGACTCGGCTTG	681-705
	2ODEAR	2nd	Reverse	TACTTCTGTTAGTGCTTTGGTTCC	3425-3402
	1ODEBF	1st	Forward	ATGATAGGGGGAATTGGAGGTTT	2388-2410
	1ODEBR	1st	Reverse	CCTGTATGCAGACCCCAATATG	5264-5243
rt-in	2ODEBF	2nd	Forward	GACCTACACCTGTCAACATAATTGG	2485-2509
	2ODEBR	2nd	Reverse	CCTAGTGGGATGTGTACTTCTGAACTTA	5219-5192
	1ODECF	1st	Forward	CAGACTCACAATATGCATTAGG	4039-4060
	1ODECR	1st	Reverse	GCCCATAGTGCTTCCTGCTGCTCCCAAGAACC	7786-7755
in-env v5	2ODECF	2nd	Forward	CTGGCATGGGTACCAGCACACAA	4146-4168
	2ODECR	2nd	Reverse	TATATAATTCACTTCTCCAATTGTC	7677-7653
	1ODEDF	1st	Forward	GAGCCAATTCCCATACATTATTGT	6855-6878
	1ODEDR	1st	Reverse	CACTCAAGGCAAGCTTTATTGAGGC	9630-9606
env v3-nef	2ODEDF	2nd	Forward	TTATTGTGCCCCAGCTGGTTTTGC	6872-6895
	2ODEDR	2nd	Reverse	GGTCTAACCAGAGAGACCCAGTACAG	9556-9531

Primers designed by Sanabani et al. (2006) are italicized, and those by Ode et al. (2015) are underlined.

* HIV-1 genomic coordinates according to the HXB2 reference (GenBank acc. # K0445).

**Fragmento A**				
SC-AOS	AGA TCT GAG CCT GGG AGC TCT CTG G	51,2	Senso	521 – 554
SC-AOA	TTT ATG GCA AAT ACT GGA GTA TTG TAT GGA		Anti-senso	2711 – 2740
SC-ANS	GCT TCA AGT AGT GTG TGC CCG TCT G	56,4	Senso	546 – 570
SC-ANA	CTT TTG GGC CAT CCA TTC CTG		Anti-senso	2590 – 2610
**Fragmento BI**				
SC-BOS	GCT AAT TTT TTA GGG AAG ATC TGG CCT T	51,8	Senso	2080 – 2107
IN-B02R	AAA TCA CTA GCC ATT GCT CTC CA		Anti-senso	4284 – 4306
SC-BNS	AGC CCC ACC AGA AGA GAG CTT	61	Senso	2157 – 2177
IN-B01R	CCA CTC AGG AAT CCA GGT GG		Anti-senso	3772 – 3791
**Fragmento BII**				
IN-B2OS	CTC ARG ACT TYT GGG AAG TTC	50,8	Senso	2800 – 2820
SC-BOA	TCT CCT GTA TGC AGA CCC CAA TAT GT		Anti-senso	5242 – 5267
IN-B01S	GAT GGG TTA TGA ACT CCA TCC TG	50,8	Senso	3772 – 3791
C-BNA	CCC TAG TGG GAT GTG TAC TTC TGA ACT TA		Anti-senso	5192 – 5220
